# Rationale, design, and methods for Canadian alliance for healthy hearts and minds cohort study (CAHHM) – a Pan Canadian cohort study

**DOI:** 10.1186/s12889-016-3310-8

**Published:** 2016-07-27

**Authors:** Sonia S. Anand, Jack V. Tu, Philip Awadalla, Sandra Black, Catherine Boileau, David Busseuil, Dipika Desai, Jean-Pierre Després, Russell J. de Souza, Trevor Dummer, Sébastien Jacquemont, Bartha Knoppers, Eric Larose, Scott A. Lear, Francois Marcotte, Alan R. Moody, Louise Parker, Paul Poirier, Paula J. Robson, Eric E. Smith, John J. Spinelli, Jean-Claude Tardif, Koon K. Teo, Natasa Tusevljak, Matthias G. Friedrich, on behalf of the CAHHM Study Investigators

**Affiliations:** 1 McMaster University, Hamilton, Canada; 2 University of Toronto, Toronto, Canada; 3 University of Montreal, Montréal, Canada; 4 Sunnybrook Research Institute, Toronto, Canada; 5 CARTaGENE, Quebec, Canada; 6 Quebec Heart and Lung Institute, Quebec, Canada; 7 Hamilton Health Sciences, Hamilton, Canada; 8 Population Health Research Institute, Hamilton, Canada; 9 Laval University, Québec, Canada; 10 Dalhousie University, Halifax, Canada; 11 McGill University, Montréal, Canada; 12 Simon Fraser University, Burnaby, Canada; 13 Alberta Health Sciences, Edmonton, Canada; 14 University of Cagary, Calgary, Canada; 15 Montreal Heart Institute, Montréal, Canada; 16 Institute for Clinical Evaluative Sciences, Toronto, Canada; 17 BC Cancer Agency and University of British Columbia, Vancouver, Canada

## Abstract

**Background:**

The Canadian Alliance for Healthy Hearts and Minds (CAHHM) is a pan-Canadian, prospective, multi-ethnic cohort study being conducted in Canada. The overarching objective of the CAHHM is to understand the association of socio-environmental and contextual factors (such as societal structure, activity, nutrition, social and tobacco environments, and access to health services) with cardiovascular risk factors, subclinical vascular disease, and cardiovascular and other chronic disease outcomes.

**Methods/Design:**

Participants between 35 and 69 years of age are being recruited from existing cohorts and a new First Nations Cohort to undergo a detailed assessment of health behaviours (including diet and physical activity), cognitive function, assessment of their local home and workplace environments, and their health services access and utilization. Physical measures including weight, height, waist/hip circumference, body fat percentage, and blood pressure are collected. In addition, eligible participants undergo magnetic resonance imaging (MRI) of the brain, heart, carotid artery and abdomen to detect early subclinical vascular disease and ectopic fat deposition.

**Discussion:**

CAHHM is a prospective cohort study designed to investigate the impact of community level factors, individual health behaviours, and access to health services, on cognitive function, subclinical vascular disease, fat distribution, and the development of chronic diseases among adults living in Canada.

**Electronic supplementary material:**

The online version of this article (doi:10.1186/s12889-016-3310-8) contains supplementary material, which is available to authorized users.

## Background

Cardiac, vascular, and cognitive dysfunction have a tremendous impact on the quality of life, longevity and health care costs in Canada, and globally. It is of paramount importance to understand the early determinants of such dysfunction and its progression to clinical events, given the increasing prevalence of known cardiovascular (CV) risk factors, which result in organ dysfunction including heart failure, non-alcoholic fatty liver disease (NAFLD), and dementia, which threatens the financial sustainability of health care systems. Cardiovascular Disease (CVD) is a leading cause of morbidity and mortality in Canada, and places a large burden of cost on the health care system. Each year approximately 70,000 Canadians die from CV causes and many more suffer life-threatening CV events such as myocardial infarction (MI) and stroke [1]. It has been estimated that cardiovascular diseases cost our health care system $22 billion dollars each year in direct and indirect costs, a figure that is expected to grow over time [2]. Additionally, CV risk factors account for up to half of the attributable risk for dementia, mediated in large part by difficult to detect microvascular disease of the brain. The rapid increase of overweight and obesity among Canadians and its associated consequences, including hypertension and diabetes add to the problem. Importantly, CVD in Canada increasingly affects women and individuals from non-white ethnic groups [1, 3]. While the treatment of clinical events caused by CVD has improved, the effective prevention of CVD with its implications on well-being and health care costs remains a challenge, due in part to knowledge gaps regarding the impact of social and built environments in relation to individual risk factors and thus on efficient political strategies to reduce CVD burden. Furthermore, there is a lack of sensitive, early risk markers and thus information on these relationships before the onset of symptomatic organ dysfunction is limited. In order to address these gaps in our knowledge, we convened the Canadian Alliance for Healthy Hearts and Minds (CAHHM) – a prospective cohort of men and women recruited through existing cohorts in Canada and an First Nations cohort.

## The specific objectives of the CAHHM are

1. To understand the role of socio-environmental and contextual factors (such as societal structure, activity, nutrition, social and tobacco environments, and access to health services) on CV risk factors, subclinical disease, and clinical CV events at the individual and population levels. This includes the impact of contextual factors on geographic variation in CVD (ie rural vs. urban, and east to west gradient), and their relative impact compared to individual level factors.
2. To characterize the unique patterns of contextual factors as well as acculturation, cultural continuity, and migration experience as related to individual CV risk factors, health service utilization (ie screening, access to diagnostics and treatments), and clinical outcomes among high risk ethnic groups including South Asian, Chinese, and African origin, as well as reserve-based First Nations people from across Canada.
3. To identify early subclinical dysfunction and tissue abnormalities in the brain, blood vessels and the heart, to characterize abdominal and pericardial fat distribution, and to investigate the association of dysfunction with contextual and individual determinants. Furthermore, the data will shed light on the predictive value of novel markers of subclinical abnormalities and dysfunction on the development of clinical events related to cardiac, vascular and cognitive dysfunction.

## Methods/Design

CAHHM is a ‘cohort of cohorts’ as the majority of participants (>80 %) will be recruited through existing cohorts: 1) Canadian Partnership for Tomorrow Project (CPTP), a harmonized longitudinal population study capturing health data, physical measures and biologics of over 300,000 Canadians (www.partnershipfortomorrow.ca). CPTP participants provide broad consent to research by internal and external scientists, and linkage to administrative health data. CPTP is a federation of five regional cohorts: the BC Generations Projects, Alberta’s Tomorrow Project, the Ontario Health Study, CARTaGENE, and Atlantic PATH (Atlantic Partnership for Tomorrow's Health), 2) the Prospective Urban Rural Evaluation (PURE)-Canada cohort, 3) the Montreal Heart Institute (MHI) Biobank, and 4) a newly formed First Nations Cohort study. The details of the separate cohorts are found in the Additional file 1.

### Eligibility and recruitment of participants into CAHHM

Participants are eligible for CAHHM if they were between ages 35 and 69 years (inclusively) at time of screening [or for First Nations participants ≥ 18 years] and willing to undergo an MRI scan and all other required study procedures. In order to recruit the majority of participants without existing CVD we asked each cohort to include participants of whom less than 20 % have known CVD, and about 50 % are women, all balanced across age strata 35–45, 46–55, 56–69 years. Some variations exist between cohorts given their differences in feasibility of recruitment and access to MRI centres. (Table 1) Details on the new First Nations cohort will be published in a separate manuscript.

**Table 1:** Participant Selection Criteria for Alliance Recruitment – By Cohort

	CARTaGENE	OHS	BCGP	APATH	Alberta’s tomorrow project	PURE	MHI	First Nations
Preliminary Selection Criteria of Participants								
Email	Yes	Yes	Yes	NA	Yes	NA	NA	NA
Blood Sample previously collected	Yes – also new sample	Yes	Yes – also new sample collected	Yes – also new sample collected	NA – obtaining new blood sample	Yes – new sample for Hamilton only	Yes – also new sample	NA – new sample
Geographic Criteria	Metropolitan Montreal and Quebec City	Greater Toronto Area, London, Hamilton, Ottawa	Metro Vancouver	Halifax and surrounding area	Calgary and Edmonton	Hamilton, Vancouver, Quebec City	Montreal	Hazelton, Maskwacis, Lac La Ronge, Sandy Bay, Fort MacKay, Thunder Bay, Six Nations, Oneida, Wendat, Pictou Landing
Prioritized Ethnic	African	South Asian, Chinese, African	East Asian, South Asian, Black	NA	NA	NA	NA	First Nations
CVD (−)	Max 20 % can have history of: MI, stroke or cancer – if not then expand	Exclude: MI, stroke, CABG, PTCA, CHF, cancer	Max 20 % can have history of: MI, stroke, CABG, PTCA, CHF or cancer	Max 20 % can have history of: MI, stroke, CABG, PTCA, CHF or cancer	Max 20 % can have history of: MI, stroke, CABG, PTCA, CHF or cancer	Max 20 % can have history of: MI, stroke, CABG, PTCA, CHF or cancer	Max 20 % can have history of: MI, stroke, CABG, PTCA, CHF or cancer	Max 20 % can have history of: MI, stroke, CABG, PTCA, CHF or cancer
Age	35–69 years at time of entry	35–69 years at time of entry	35–69 years at time of entry	35–69 years at time of entry	35–69 years at time of entry	35–69 years at time of entry	35–69 years at time of entry	18 years and up
Additional Sampling after above criteria considered								
Participant Selection	Random	Random	Random	Random	Random	Consecutive	Consecutive	Volunteer
Cohort specific selection criteria	Prioritized for existing RNA sample and genomic information					Approach participants coming for in-person follow up visit		
Exclusions	Participants in the Diabetes/Depression substudy or with pacemaker				Recently invited to participate in other sub-studies			
Log of participant kept or available	Yes	Yes	Yes	Yes	Yes	Yes	Yes	NA

### Clinical assessment

The clinical assessment for CAHHM participants consisted of: a) completion of questionnaires, b) physical measurements, c) collection of blood samples in some participants (stored blood samples will be used for others), and d) a MRI scan of brain, heart, carotid artery and abdomen, details of each component are provided below.

### Health questionnaires

Personal and Medical History were collected using standardized questions including family history, and health behaviors. Diet and physical activity were collected using a food frequency questionnaire (FFQ) and the short form International Physical activity Questionnaire (IPAQ). (Table 2)

**Table 2:** List of Measures/Questionnaires of Baseline Visit

Questionnaire/Measure	Source	Time to complete (minutes)	Method
Physical Activity	IPAQ-S [71]	4	Self Administered
Dietary Intake (Macro and Micro Nutrients)	SHARE-FFQ [72] and DHQ II	22	Self Administered
Cognitive Function: Digital Symbol Substitution	DSS [73]	5	Administered by RA
Montréal Cognitive Assessment	MoCA [9]	8	Administered by RA
Community Factors (Individual Perception)	EPOCH-2 [58]	21	Self Administered
Immigrant Questionnaire	Longitudinal Survey of Immigrants in Canada [60]	3	Self Administered
Acculturation	Vancouver Inventory of Acculturation [61]	3	Self Administered
CVD related Health Services Questionnaire	Multi-source: Canadian Community Health Survey, Ontario Health Study	7	Self Administered
General CVD Questionnaire	Prior Longitudinal Studies (SHARE, PURE)	7	Self Administered
CPTP Core Baseline Health and Lifestyle Questionnaire	CPTP Study [74]	14	Self Administered
Physical Measures (blood pressure and heart rate via OMRON cuff, Body Fat & Weight via Tanita BIA)		10	Clinic

RA: Research Assistant

### Cognitive function

Cognitive function measures in CAHHM were selected balancing the need for a brief, cost effective, and sensitive measure appropriate for use in a 35–69 year age group. Two assessments were chosen, first the Digit Symbol Substitution (DSS) test (Wechsler Adult Intelligence Scale IV version) was chosen because it displayed age-related effects over the age range of 40–70 years in participants in the PURE-MIND study, a contemporary Canadian-based prospective cohort study of 800 participants with similar entry criteria to the Canadian Alliance for Healthy Hearts and Minds [4]. In the DSS, the participant transcribes as many coded symbols as possible within a given time (in this case, two minutes). Lower scores indicate worse performance. The DSS is sensitive to change over time [5], is lower in persons with silent brain infarct, is independent of language, is sensitive to mildly impaired cognition [6], and predicts clinically important events such as falls and mortality [7, 8]. Second, the Montréal Cognitive Assessment (MoCA) was used as a global cognitive screening test. The MoCA takes 10–15 min to administer and briefly evaluates the following domains: delayed recall, verbal fluency, visuospatial skills, clock drawing, executive functions, calculation, abstraction, language, orientation, attention and concentration [9]. The test has a sensitivity of 90 % and specificity of 87 % to detect mild cognitive impairment in patients and distinguish them from normal controls [10]. The MoCA has been validated in memory clinic settings for diagnosis of mild cognitive impairment or dementia [9–13]. The MoCA tests domains including executive function and therefore should be more sensitive to vascular cognitive impairment than the Folstein Mini mental status examination [14–18], is more sensitive to milder forms of impairment [9, 15, 19] and shows less ceiling effect [19, 20].

### Physical measurements

Participants height, weight, percent body fat using the Tanita BIA machine, waist circumference, hip circumference, resting heart rate, and blood pressure using an automated OMRON cuff are collected. All measurements are taken using a standardized protocol.

### Blood collection

New blood samples are collected from cohorts in which blood had not previously been collected from their parent cohort. A new blood sample will be collected from First Nations participants, the Alberta’s Tomorrow Project, BC Generations, CARTaGENE, Atlantic PATH, MHI Biobank, and PURE (Hamilton site only).

### Rationale for MRI imaging

Imaging-derived markers can be acquired non-invasively and include focal tissue, thereby increasing the sensitivity to detect vascular lesions and tissue pathology with prognostic impact. Among the available imaging tools, MR imaging combines an outstanding safety profile with excellent accuracy and reproducibility. Its advantages also include a high spatial resolution, a small observer dependence, and excellent. Moreover, it allows for a comprehensive, multi-target approach including morphology, mass, function, flow, vessel lumen, tissue composition, and metabolism. It can be considered the most efficient imaging tool for clinical or cohort studies in healthy individuals. Several existing and conceptualized population-based cohorts utilize either cardiac or brain MRI, including the UK BioBank [21], The German National Cohort study [22], the Dallas Heart Study [23] the MESA study [24], Age, Gene/Environment Susceptibility –Reykjavik Study [25, 26], Framingham study [27, 28] and Rotterdam study [29, 30]. However, few studies have comprehensively assessed the cardiovascular system using MRI, including arterial imaging. In CAHHM, participants will undergo a comprehensive MR examination of the brain, heart, carotid artery and abdomen (for visceral and liver adiposity). Of note, the CAHHM not only uses markers with demonstrated value, but also novel candidates related to coronary plaque stability and microvascular function.

### MRI Protocol

Details of the CAHHM MRI protocol are found in Tables 3 and 4. The protocol was developed in collaboration with a multi-disciplinary expert group (see working groups listed at the end of the conclusion section) balancing the scientific objectives with time efficiency. The protocol uses validated standard techniques and provides information on morphology, function and tissue characteristics. Quantitative data are measured or calculated wherever possible. Aside from established parameters, novel markers are included, with most of them being acquired by additional images as part of an extended protocol, performed with the MR contrast agent gadobutrol.

**Table 3:** MRI key measures in Heart, Brain, Abdomen during Standard MRI Protocol

Standard		
Sequence	Approximate Imaging Time (min)	Outcome Measure
Cardiac		
2D Cine SSFP (short axis only)	10	LV global (EF) and regional wall motion abnormalities function
		LV mass index
		LV end-systolic volume index
		LV mass-to-volume ratio
		LA size and function
		RV volume and global function
Brain		
3D T1w MPRAGE	8	Brain Volume
2D Flair	3	Covert stroke and white matter lesion burden
Abdomen		
T1w TSE abdominal adipose tissue sequence	2	Visceral fat area
Liver 2D multi-echo gradient-echo sequence	1	Liver fat %
Cerebrovascular		
3D T1w MPRAGE	6	Plaque volume and intraplaque hemorrhage detection
TOF	6	Plaque volume and intraplaque hemorrhage detection
Additional time for positioning		
Coil Positioning	6	
Total time	42^a^	

^a^Note this varies depending on: Scanner make and model, hardware and software used for scanning, need for switching MR coils, participants heart rate, and MRI technologist experience

**Table 4:** Extended MRI Scan Protocol

Extended		
Sequence	Approximate Imaging Time (min)	Outcome Measure
Cardiac		
Cine SSFP (3 long axis views and 10–15 short axis views)	12	LV global (EF) and regional wall motion abnormalities function
		LV mass index
		LV end-systolic volume index
		LV mass-to-volume ratio
		LA size and function
		Circumferential strain
		RV volume and global function
Phase-contrast cine	4	Aortic elasticity
LGE	12	Myocardial fibrosis
T1 Mapping	10	Diffuse fibrosis
T2 star-weighted sensitive sequence	10	Microvascular function
Brain		
3D T1w MPRAGE	8	Brain Volume
PD/T2	4	Covert stroke and white matter lesion burden
T2 star-weighted gradient echo sequence	4	Presence of microbleeds
2D resting state fMRI	4	Functional connectivity
3D arterial spin labeling (ASL)	7	Cerebral blood flow
2D diffusion tensor imaging (DTI)	5	White matter connectivity
Abdomen		
T1w TSE abdominal adipose tissue sequence	2	Visceral fat area
Liver 2D multi-echo gradient-echo sequence	1	Liver fat %
Cerebrovascular		
3D T1w MPRAGE	8	Plaque volume and intraplaque hemorrhage detection
TOF	6	Plaque volume and intraplaque hemorrhage detection
3D T1w MPRAGE + contrast	5	Lipid core, calcification inflammation/angiogenesis
Additional time for positioning		
Coil Positioning	6	
Biobreak	10	
Total time	118	

### Cardiac dysfunction

Cardiac MR (CMR) will provide data on global and regional left ventricular and right ventricular function. This rationale is based on data from the MESA cohort with its multi-ethnic population-based sample of 4510 individuals (mean age 61y), where a 25.6 % prevalence of regional wall motion abnormalities (RWMA), with a 2.6 % heart failure rate (vs. 1.0 % in individuals without RWMA) was observed [31]. With its obvious potential as a predictor for heart failure, the correlation of RWMA with other risk markers and especially environmental factors becomes an important target for preclinical research. Its standardized acquisition [32, 33], high prevalence in the general population and strong predictive value for subsequent heart failure render CMR-derived RWMA an excellent early marker and primary endpoint in CAHHM. Other markers include myocardial scarring [34, 35], and coronary vascular function [36–38]. Furthermore, this is an unprecedented opportunity to acquire multi-ethnic normal values for numerous quantitative cardiac markers. Because of its outstanding safety profile, standardized approaches, accuracy, reproducibility and comprehensive multi-parametric protocols, we considered CMR the most useful tool for detecting subtle, early and local changes which may precede cardiac, vascular and cognitive dysfunction.

### Covert stroke and cognitive dysfunction

Recent population-based studies have shown that the prevalence of unrecognized “covert” brain infarcts is as high as 30–40 % in the elderly/geriatric population, and current evidence is sufficient to document that covert strokes and ischemic white matter damage in the elderly are associated with cognitive impairments despite the lack of association with specific symptoms, and are highly predictive of future stroke and dementia. However, important gaps in knowledge include the prevalence of brain infarcts prior to age 60 (as previous studies have focused on the elderly), the predictive power of covert stroke and ischemic white matter damage as a marker of important CV events in addition to future stroke (such as myocardial infarction), and the relationship between covert stroke and subclinical manifestations of CVD in other organs. Additionally, both covert stroke and ischemic white matter damage are partly heritable but the genetic basis of this risk has not been well defined yet.

Emerging evidence suggests that alterations in brain structure and function accrue years before clinical symptoms of stroke or cognitive dysfunction. In Alzheimer’s disease, regional brain atrophy and altered glucose metabolism can be detected on neuroimaging 15 years before the onset of symptoms [39]. By contrast, the changes in brain structure and function associated with CV and cerebrovascular disease are largely unknown, because previous population-based studies used last-generation MRI technology without high resolution imaging. We expect that changes in brain structure (eg as measured by volumetric brain MRI) and brain functional and structural connectivity (eg as measured by resting state functional MRI and diffusion tensor imaging of white matter tracts) can be detected in association with CVD such as hypertension and diabetes, both in the presence and absence of MRI-visible signs of irreversible ischemic damage (that is, covert stroke and ischemic white matter damage). This hypothesis will be addressed in the Alliance for Healthy Hearts and Minds, in which high-resolution brain MRI imaging and a comprehensive assessment of CV risk are collected.

### Carotid atherosclerosis

Subclinical atherosclerosis detected through imaging of the carotid arteries can provide insights into the type and burden of atherosclerotic disease. Prior studies have shown that the carotid vascular bed also acts as a good representative of vascular disease throughout the body providing a ‘vascular phenotype’ for the individual [40]. A variety of imaging techniques are available for carotid artery imaging, for instance Ultrasound generated intimal medial thickness (IMT) and 3D ultrasound. These two sequences will provide quantitative data regarding luminal narrowing and vessel wall volume to detect carotid plaque. In addition, the vessel wall imaging is able to characterize plaques as high or low risk by detecting the presence or absence of plaque hemorrhage [41]. Among the subset of participants undergoing the extended scan in which intravenous gadolinium is administered plaques can be further characterized for the presence of lipid within the plaques.

### Measuring ectopic fat deposition in CAHHM

Although several imaging studies have documented significant associations between measures of ectopic fat accumulation (including visceral adiposity), cardiometabolic risk markers and clinical outcomes, most of these large cohort studies (Framingham [42–48], Jackson Heart Study [49–51], MESA [52, 53], INSPIRE ME IAA [54], CARDIA [55, 56]) have used computed tomography to quantify abdominal subcutaneous and visceral adiposity as well as the accumulation of unwanted lipid deposition in normally lean tissues such as the heart, the liver, the pancreas and perivascular adipose tissue. Furthermore, among studies using MRI such as the Dallas Heart Study, the Chengdu Study and the NEO Study, most of them have been limited in scope and do not cover the comprehensive spectrum of outcomes considered in CAHHM. The present study will therefore be one of the most comprehensive cardiometabolic MRI studies ever conducted and will provide a unique opportunity to decipher the respective contributions of specific ectopic fat depots to a plethora of clinical conditions, going way beyond various cardiovascular outcomes including particular attention to several indices of brain function and health. Furthermore, the substantial subgroup of First Nations people will generate very much needed data for that population for whom we do not currently have adequate imaging data to properly describe to what extent they are susceptible or not to ectopic fat deposition.

### Contextual factors measured at the individual and community level

Data describing contextual factors that characterize the nutrition, physical and tobacco environments of communities from which participants are recruited as well as individual behaviors in these domains are collected using the modified EPOCH-1 and modified EPOCH-2 questionnaires modified from the PURE study [57, 58]. EPOCH-1 is a standardized community audit developed and validated in the PURE study [57] and EPOCH-2 captures individual’s perceptions of their food, activity, and tobacco environment also developed in the PURE study [58] with added questions on social ties, alcohol use, and workplace activity and food choices and behaviors.

### Community audits

To study community level contextual factors as related to individual risk factors and clinical events we retrospectively defined “community” as participants are already recruited. After review of the communities from which participants in the 5 CPTP cohorts and the 2 partner cohorts originate, the forward sortation areas (FSA) was deemed to be the optimal community unit. (Table 5) We have chosen the FSA as our definition of community because: 1) there was low representation of cohort participants from census tracts in rural areas and eastern provinces of Canada, 2) the FSA are those reported by census respondents for their place of residence and this information collected from the census is available in aggregate for each FSA. This census information includes age, sex, marital status, families and household information, housing costs, mobility and migration, immigration and citizenship, income and earnings, and ethnic groups. This is balanced by some limitations of this approach include: for certain highly populated urban areas the FSA is too large to well represent the community—in this scenario, we surveyed multiple postal codes within the FSA to capture the diversity. The community level information will be used together with self-reported perception of community environment as well as behavior patterns, ie shopping, activity, and workplace. These will be used together with objective measures of the built environment from publically available databases (ie streetsmart walk-score at www.walkscore.com), which provides information by postal code on neighborhood walkability, land use mix, transportation availability and location of food retail outlets [59].

**Table 5:** Number of FSAs represented by the 7 Cohorts making up the CAHHM

Province	FSA total	FSA to be assessed in CAHHM (Postal Codes)*	# in reliability assessment (%)	Number of postal codes within FSAs being assessed (sum)
BC	190	190 (+50)		112904
AB	153	153 (+46)	40 (21.1 %)	76924
SK	49	49 (+8)		21541
MN	66	66 (+12)		23943
ON	526	526 (+208)		282123
QC	419	419 (+108)	55 (10.4 %)	215565
Atlantic	230	230 (+72)		92809
NS	77	77 (+26)		28171
NB	111	111 (+30)		59530
PEI	7	7 (+2)		3995
N/L	35	35 (+14)		1113

*For FSA with income discrepancies across postal codes within the FSA, 2 audits were done to reflect the 25^th^ percentile an

### Social capital and social ties

A series of validated questions were used to measure social support from the family and the wider social environment and included questions such as participation in community organizations, civic engagement, perceived social standing, and intensity of social relationships with close confidants, and type of contact with confidants (ie in-person, telephone, email, Facebook, text messages).

### Culture and immigration

Among immigrants to Canada we will probe the reasons for immigration (economic, family, refugee) and the socio-cultural connections they have made since immigration using the Immigration Questionnaire [60] and the Vancouver Inventory of Acculturation [61]. This information will be used along with measures of socioeconomic status (education, household income, employment and marriage) which has already been collected in each of the participating cohorts.

### Access to and quality of health care services

A health services questionnaire was developed to collect information at the individual level and will be supplemented by record linkage to administrative, laboratory and clinical databases available in various Canadian provinces. With these data sources we plan to analyze existing population-based databases to create unique community-level profiles of access and quality of health care services including the rates of selected CV related diagnostic tests and treatments. This will be a unique component of this initiative because health care services have not been a focus of most traditional cohort studies to date and yet, it is increasingly recognized that quality of health care provided to individuals can play a major role in determining their likelihood of suffering and surviving clinical events [62]. Furthermore, how Canadians with CV risk factors are managed likely varies across the country due to factors such as physician and allied health care personnel availability, type and nature of primary care services, patient education, and socioeconomic status.

### Inclusion criteria

1. Participants between ages 35 and 69 y (inclusively) at time of screening
2. Provision of Informed Consent
3. The participant is willing to undergo an MRI scan

### Exclusion criteria

1. Participant is claustrophobic and/or is known to suffer from moderate to severe anxiety during MRI scans or similar procedures
2. Participant is obese and/or exceeds equipment weight limit and/or circumference of the MRI portal at time of screening
3. Participant has a metallic implant or another foreign body which is not compatible with Magnetic Resonance Imaging (MRI) (eg pacemakers, defibrillator, vascular clips, drug pumps, implant(s), or any other foreign bodies, extensive tattoo covering a large part of their chest or head)
4. Female participants that are or may be pregnant (confirmed or uncertain)
5. Received an MRI contrast agent within 72 h prior to the MRI scan.

#### Record linkage with health administrative databases

Data collected of participants from the CAHHM will be linked with health care administrative databases, available in various provinces, for ascertainment of cardiovascular-related health care services before and after enrollment in the study as well as long-term CVD outcomes. (Table 6) The International Classification of Diseases (ICD) codes that will be used to measure various outcomes have been validated and described in detail elsewhere [63]. Most provincial health care administrative databases in Canada contain a patient health card number as a unique identifier that enables efficient deterministic data linkage of various data sets, in this instance cohort data sets with province- or region-specific administrative databases. To protect patient privacy, the health card number (HCN) in each database is typically scrambled creating another unique anonymized identifier before the actual data linkage. All participants were asked on the informed consent if they were willing to provide HCN as part of their parent cohort participation or as a new request (and thus far out of the first 1000 participants 99.2 % have consented to provide their HCN). Due to the federated nature of the health care system in Canada, there is no single repository of administrative databases. As a result, data linkage activities will involve working with multiple data custodians and governments across the country, and will require understanding of and compliance with each province’s health information privacy legislation. The Alliance is planning to work with key data custodians such as the Canadian Institute for Health Information (CIHI), which collects hospital discharge abstracts for the entire country except for Quebec, Statistics Canada which houses the Canadian Mortality Database and the Canadian Cancer Registry and various provincial health services research units located across Canada including the Institute for Clinical Evaluative Sciences (ICES) in Ontario, Population Data BC, the Population Health Research Unit, Dalhousie University, Régi de l’assurance maladie Québec (RAMQ), etc. - each of which hold administrative databases for their region/province.

**Table 6:** Health system quality indicators

Health system quality indicators measurable from the CPTP CVD Survey
Participants with a family doctor
Difficulties accessing primary or specialist care
Weight assessment by a health professional
Screening for hypertension, diabetes, hyperlipidemia
Diabetics who have had eyes examined by an ophthalmologist, feet examined, urine protein tested
Smokers who have been offered smoking cessation counseling and/or stop smoking aids
Hypertensives who have their blood pressure treated and controlled
Participants with hyperlipidemia who have been treated with medication to control their blood cholesterol levels
Participants with atrial fibrillation who have been treated with blood thinners
Health system indicators measurable from administrative data
A. Structural variables
# of family doctors/specialists per capita
#, costs and types of ambulatory care visits
#, costs and types of hospitalizations
B. Processes of Care
CV and non-CV medications (statins, ACE inhibitors, diuretics, Beta-blockers)
Laboratory screening rates (lipids, diabetes)
Lab results (lipids, diabetes)
ECG, Stress Test, Echo, CT scans, MRI scans
Cardiac Catheterization
PCI
Cardiac Surgery
C. Outcomes
Myocardial Infarction (STEMI/NSTEMI, unstable angina)
Congestive Heart Failure
Stroke (Ischemic/Hemorrhagic/TIA)
Death (including cause of death)
Atrial Fibrillation
Diabetes and Hypertension

### Study outcomes

#### Primary outcomes

The primary outcomes include MI, Stroke, angioplasty, percutaneous coronary interventions, coronary artery bypass graft surgery, and other important chronic disease outcomes and death. Associations between contextual factors, CV risk factors and MRI markers will be evaluated with these outcomes individually and as a composite measure of CV events.

#### Secondary outcomes

Secondary outcomes for this project include:

- Congestive Heart Failure requiring hospitalization

#### New onset established risk factors using validated alogrithms [63, 64]

- Incident diagnosis of diabetes by physician
- Incident diagnosis of hypertension by physician
- Incident diagnosis of significant cognitive dysfunction (ie dementia) by physician.

#### Risk markers acquired through imaging and blood samples

- Acquired parameters that are linked to the present health status
- Candidate parameters for predicting cardiovascular events which affect cardiac and cognitive dysfunction. For further details, please refer to the outcomes in the questionnaires and the MRI protocol described in the Design and Methods section above.

## Statistical considerations

### Power

#### Risk factor proportions

The proportion of risk factors will be examined across cohorts and overall. It is anticipated given the roughly representative subcohorts that recruitment of the target sample of 7000 will provide high power to compare the relative frequency of risk factors comparing men and women, participants across age strata and between non-First Nations and First Nations' Participants.

#### Risk factors to MRI findings

The prevalence of traditional CV risk factors will enable determination of the relative risk of the risk factors on subclinical MRI findings of RWMA, covert stroke and liver adipose tissue. For example, if among 7000 CAHHM participants the frequency of hypertension is 25 % and the frequency of covert stroke is 6.6 %, we will have a high power to detect a relative risk of hypertension on silent stroke of brain of 1.5 (95 % CI: 1.36 to 1.65).

#### MRI to clinical events

Several MRI markers have been tested for their predictive value versus CV events in several populations. Given the planned 7000 subjects and a predicted incident CV 5 year event rate of 5.98 %[1], we have high power (ie >90 %) to detect a hazard ratio (HR) of 2.6 among those with RWMA by MRI, high power to detect a HR of 2.85 for stroke among those with silent stroke by MRI. Further we will have approximately 80 % power to detect a 1.6-fold increase in new diabetes in those with liver fat as estimated by MRI assuming an incidence of new diabetes of approximately 10 %. Estimations of power for sample sizes lower than this (ranging from 5000 to the anticipated 7000 subjects) are also shown in Table 7. Given the limited information regarding the predictive relationship between liver fat and CVD, we identified only one published study conducted in diabetics which suggested that liver fat was associated with a 1.9-fold increase in CV incidence [65], on the other hand a study by Lazo et al. [66] reported that NAFLD by ultrasound was not associated with excess in all-cause or cardiovascular mortality in the NHANES III study – general population. While our power is too low to detect a HR of 1.9 between liver fat and CVD, our multiethnic sample which includes 2 high risk groups for visceral fat and especially liver fat and in whom we are using a superior measure of liver fat (which has greater precision as compared to ultrasound) [67] provides an opportunity for us to test the association between liver fat and CVD events in otherwise understudied populations.

**Table 7:** Power of given sample size between 6400 and 7400 participants recruited to detect expected hazard ratios

Exposure	Assumed prevalence of exposure	Outcome	Assumed incidence of Ooutcome	Expected hazard ratio	7000/ (5920)^1^	6000 /(5120)^1^	5000/(4320)
Regional Wall Motion Abnormality	0.253	CVD	0.0598	2.60	>0.99/>0.99	>0.99/0.99	>0.99/>0.99
Silent Stroke	0.069	CVD	0.0598	2.85	>0.99	>0.99	>0.99
Liver Fat^a^	0.063	T2DM	0.0974–0.1275	1.60	0.82	0.76	0.68

^1^ Assuming 20 % of the participants will have existing CVD the Number of Participants free of CVD and Cancer are shown in parenthesis; ^a^ (assuming a higher proportion of First Nations participants are retained, relative to other groups)

#### Analysis

To investigate the association between risk factors and subclinical MRI findings linear (visceral fat) and logistic (RWMA, covert stroke, liver fat %) regression models must be built separately for each MRI outcome used as the dependent variable. Exposures such as age, sex, ethnicity, history of hypertension, diabetes, waist circumference, apolipoprotein B/A ratio, current smoking, physical activity, selected dietary (ie prudent diet score, or ratio polyunsaturated fat/saturated fat), and measures of cognitive function (ie when covert stroke is the outcome) will first be tested univariately, and exposures with a *P* < 0.10 will be taken forward and tested in multivariate regression models. If ethnicity is found to be an independent predictor of an MRI outcome, it will be tested with a composite CV risk score for an interaction with the outcome.

After 5 years, when enough CVD events have been accrued and ascertained through record linkage, the MRI subclinical exposure of covert stroke, global or regional ventricular function abnormalities, liver fat and visceral fat will be tested versus CVD and death to determine their predictive value of clinical events using the area under the curve (C statistics), and net reclassification improvement methods [68].

#### Contextual factors

Comparisons between urban and rural communities will be conducted by linear mixed models for continuous variables (ie BMI) and generalized linear mixed model (GLMM) l for categorical variables (ie CVD events), in each case treating community as the random effect. Using a similar model, unadjusted correlations will be estimated between the perceived (EPOCH-2) and objective environmental measures (EPOCH-1) with the continuous or categorical outcomes. Multilevel modeling will be used to evaluate the relation between exposures at the community (FSA-level) and individual level with outcomes for each individual. All models will examine both perceived and objective environmental measures as covariates adjusted for individual and community socio-demographics. Interaction terms between urban and rural and the perceived and objective environmental measures will be investigated to determine if the contextual factors differ based on the type of community ie urban or rural. The contribution of variance in outcomes explained by community risk factors as opposed to the individual risk factors will be quantified using the variance partition coefficient from each model. For the continuous outcomes, linear multi-level modeling will be used. For categorical outcomes (ie CVD), similar generalized linear multi-level models will be fit. Models will be adjusted a priori for individual (age, sex, ethnicity, and household income) and community variables.

#### Data management and data access

Participants provide information through self-reporting (eg Health Services Research - HSR, Food Frequency Questionnaire - FFQ) and interviewer administered tests (eg cognitive function testing). Participants are invited by email/mail and then directed to provide a brief informed consent and complete 2 questionnaires on-line (HSR, FFQ), once booked for an MRI visit and screened for contraindication to MRI, they complete the remainder of the assessment at the MRI site prior to or immediately after their MRI. Physical measurements are taken by clinic staff following a standardized protocol. Participants are given a card or self–record their physical measurements, highlighting any abnormal results. Data is then entered at the clinical site or faxed in using Datafax. Quality control notes are sent by the coordinating centre to the sites to correct any missing or abnormal values.

### Results reporting

#### CV risk score

All participants receive a CV risk score report after their baseline assessment. The majority of participants receive the non-lab INTERHEART risk score which includes age, sex, family history, diabetes, hypertension, diet, activity, smoking, second hand smoke and psychosocial factors as previously described [69], and the First Nations participants receive the lab-based risk score which include apolipoproteins A and B and HbA1c..

### Incidental findings (CIF)

**Fig. 1:**
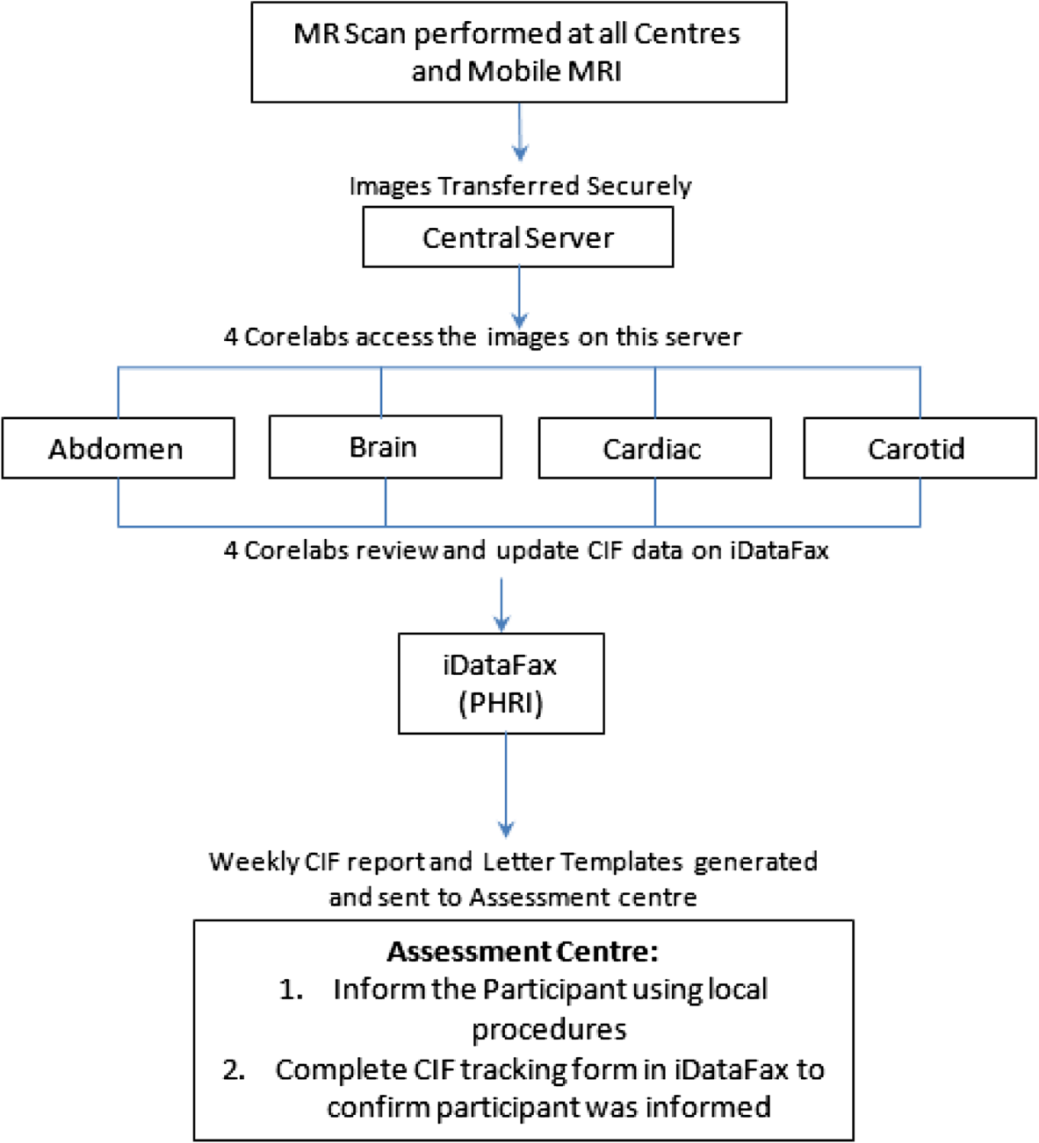
MRI analysis and Clinical incidental Findings reporting

Four core labs separately assess the Brain (Calgary or Sunnybrook), Cardiac (Montreal Heart Institute), Carotid (Sunnybrook) and Abdomen (IUCPQ). The readers follow a standardized reading protocol. Results are sent to the central project office, where they are linked with the clinical data. These severe structural abnormalities are reported back to participants and their primary care physicians if they consented to this on their informed consent. (Fig. 1) The severe structural abnormalities are shown in the Table 8. The letter emphasizes that this is a research scan which should be followed up with a targeted clinical scan organized by the primary care physician.

**Table 8:** Severe Structural Abnormalities

Abnormality	Criteria
Brain infarct	Diameter ≥15 mm or cortical location
Myocardial infarction	High signal in LGE images from extended MRI scan or a segmental wall thickening of <10% (severe hypokinesis/akinesis) for at least 1 of the 16 standardized segments.
Aortic dilatation	Thoracic ˃50 mm (men), ˃45 mm (women)
	Abdominal ˃45 mm (men), ˃40 (women)
Valvular dysfunction	Moderate or severe, with LV dilatation or dysfunction
Mass	Positive criteria for malignancy or significant compression or infiltration of vital structures

## Discussion

The Canadian Alliance for Healthy Hearts and Minds seeks to understand the individual and contextual origins of CVD risk and will aid in the design of effective policy and health interventions aimed at reducing population levels of risk factors in Canada. CAHHM is a unique cohort study which brings together participants’ enrolled in diverse cohorts from chronic disease focused cohort studies, in addition to creation of a new First Nations cohort.

First, CAHHM is unique as MRI is being used to identify subclinical disease, and is only one of two studies we are aware of interrogating the brain, heart, carotid arteries, and abdominal adiposity [70]. MRI represents an advantage over other imaging modalities as it is sensitive to detect early, subclinical stages of disease on a systematic (blood) or regional (tissue) level. Availability of such validated predictors may allow the development of more effective early treatment and personalized primary prevention strategies.

Second, we developed a detailed health services questionnaire which collects information on primary care visits, counseling for health behaviors, screening for risk factors, access to pharmacists, and visits to specialist physicians. To our knowledge such a detailed collection of cardiovascular-related health services information has not been collected on such a large cross section of Canadians and will provide valuable information to enable creation of a Health Services “Report Card” for Canadians in urban and rural/remote regions.

Third, our contextual assessment of communities across Canada together with data from individuals included in our partner cohorts will enable an investigation of the “causes of the causes”, specifically the influence of contextual factors on CV risk factors. Two contextual factor assessments are being undertaken. The modified EPOCH-1 is a standardized audit completed by research personnel at the FSA level in all provinces of Canada and First Nations reserves. This will provide information on the tobacco environment, nutrition environment, activity environment, and social connectedness. Their precise characterization in communities across Canada will aid policy development and inform population health interventions aimed at reducing the risk for cardiac and cognitive dysfunction, and other chronic diseases.

Finally, we strive to establish a diverse cohort. Specific strategies we have adopted to enhance diverse ancestry recruitment include: i. establishment of a new First Nations cohort recruited from 10 communities across Canada, ii. prioritization by ethnicity ie South Asian, Chinese and African origin participants in the CPTP cohorts’; and iii. targeted ethnic recruitment for South Asians and Chinese origin people in Ontario.

### Anticipated challenges

The CAHHM initiative is a massive undertaking to coordinate the recruitment of new and existing cohort participants from 7 existing cohorts and a new First Nations cohort, facilitating MRI scanning in urban, rural and remote regions, and conducting record linkage for clinical events using Health Card Number, across provinces, at multiple future time points. However, the study is robustly underway due to the strong commitment of interested participants, researchers, academic institutions, and funding agencies.

## Conclusions

The Canadian Alliance for Healthy Hearts and Minds is a prospective cohort being established in Canada with unique features including recruitment of individuals from existing cohort studies, use of MRI of brain, heart, carotid artery and abdomen to detect subclinical abnormalities, detailed measurement of health services utilization, and measurement of individual and community level contextual factors. The information generated in CAHHM will be used to develop community and individual level CV prevention strategies for the people of Canada.

### CAHHM coordination and working groups

#### Central coordination

Population Health Research Institute: Project Manager: Dipika Desai; Study Coordinator: Melissa Thomas; Research and Data Management Assistants: Sherry Zafar, Shaathaka Nandakumar, Sheila Bouseh, Natalie Campbell. Finance and Contracts: Beena Cracknell, Tanya Chow, Inosha Witharana, Colin Russell; Information and Communication Technology: Kevin Archibald, Kristen Avery; Statistics and Biometrics Programming: Karleen Schulze, Xiumei Yang, Cissy Tang

**Advisory Committee:** Pierre Boyle, Jean Rouleau, Eldon Smith, Caroline Wong.

**Magnetic Resonance Imaging Coordination:** Montreal Heart Institute: Felipe Henriques, François Marcotte, Julie Lebel, Matthias Friedrich.

**Health Services/Record Linkage Coordination:** Jack Tu, Natasa Tusevljak, Laura Maclagan.

**CPTP National Coordinating Centre:** Jacques Magnan, Celine Moore.

**Imaging Working Group**: Richard Frayne, Cheryl McCreary, Eric Smith, Sandra Black, Alan Moody, Christopher Scott, Jean-Pierre Despres, Eric Larose, General Leung, Tarik Hafyane.

**Health Services Working Group**: Finley McAlister, Nadia Khan, Jafna Cox, Dennis Ko, Douglas Lee, Louise Pilote, Jack Tu.

**Contextual Working Group**: Sonia Anand, Joseph Beyene, Gillian Booth, Daniel Corsi, Russell de Souza, Lise Gauvin, Scott Lear, Ayesha Rana, Fahad Razak, SV Subramamian, Jack Tu.

**First Nations Working Group**: Sonia Anand, Ellen Toth, Sharon Bruce, Stewart Harris, Christopher Lai, Paul Poirier, Sylvia Abonyi, Heather Castleden, James Irvine, Diana Lewis, Laura Arbour.

**Ethnic Working Group**: Maria Chiu, Gordon Moe, Jack Tu, Sonia Anand.

**Data Harmonization and Ethics:** Isabel Fortier, Bartha Knoppers, Ma’n Zawati.

## Additional file

Additional file 1: CAHHM Local Site Investigators and MRI Coordination. CAHHM Participating Cohorts PIs. (DOCX 12 kb)

## References

[1] Tu JV, Nardi L, Fang J, Liu J, Khalid L, Johansen H (2009). Canadian Cardiovascular Outcomes Research T: National trends in rates of death and hospital admissions related to acute myocardial infarction, heart failure and stroke, 1994–2004. CMAJ.

[2] Smith ER (2009). The Canadian heart health strategy and action plan. Can J Cardiol.

[3] Tu JV, Chu A, Rezai MR, Guo H, Maclagan LC, Austin PC, Booth GL, Manuel DG, Chiu M, Ko DT et al. The Incidence of Major Cardiovascular Events in Immigrants to Ontario, Canada: The CANHEART Immigrant Study. Circulation. 2015; 10.1161/CIRCULATIONAHA.115.015345 PMC4606988 26324719

[4] Smith EE, O’Donnell M, Dagenais G, Lear SA, Wielgosz A, Sharma M, Poirier P, Stotts G, Black SE, Strother S (2015). Early cerebral small vessel disease and brain volume, cognition, and gait. Ann Neurol.

[5] MacDonald SW, Hultsch DF, Strauss E, Dixon RA (2003). Age-related slowing of digit symbol substitution revisited: what do longitudinal age changes reflect?. J Gerontol B Psychol Sci Soc Sci.

[6] Proust-Lima C, Amieva H, Dartigues JF, Jacqmin-Gadda H (2007). Sensitivity of four psychometric tests to measure cognitive changes in brain aging-population-based studies. Am J Epidemiol.

[7] Fried LP, Kronmal RA, Newman AB, Bild DE, Mittelmark MB, Polak JF, Robbins JA, Gardin JM (1998). Risk factors for 5-year mortality in older adults: the Cardiovascular Health Study. JAMA.

[8] Rosano C, Newman AB, Katz R, Hirsch CH, Kuller LH (2008). Association between lower digit symbol substitution test score and slower gait and greater risk of mortality and of developing incident disability in well-functioning older adults. J Am Geriatr Soc.

[9] Nasreddine ZS, Phillips NA, Bedirian V, Charbonneau S, Whitehead V, Collin I, Cummings JL, Chertkow H (2005). The Montreal Cognitive Assessment, MoCA: a brief screening tool for mild cognitive impairment. J Am Geriatr Soc.

[10] Smith T, Gildeh N, Holmes C (2007). The Montreal Cognitive Assessment: validity and utility in a memory clinic setting. Can J Psychiatry.

[11] Damian AM, Jacobson SA, Hentz JG, Belden CM, Shill HA, Sabbagh MN, Caviness JN, Adler CH (2011). The Montreal Cognitive Assessment and the mini-mental state examination as screening instruments for cognitive impairment: item analyses and threshold scores. Dement Geriatr Cogn Disord.

[12] Freitas S, Simoes MR, Alves L, Santana I (2013). Montreal cognitive assessment: validation study for mild cognitive impairment and Alzheimer disease. Alzheimer Dis Assoc Disord.

[13] Larner AJ (2012). Screening utility of the Montreal Cognitive Assessment (MoCA): in place of--or as well as--the MMSE?. Int Psychogeriatr.

[14] Dong Y, Sharma VK, Chan BP, Venketasubramanian N, Teoh HL, Seet RC, Tanicala S, Chan YH, Chen C (2010). The Montreal Cognitive Assessment (MoCA) is superior to the Mini-Mental State Examination (MMSE) for the detection of vascular cognitive impairment after acute stroke. J Neurol Sci.

[15] Hachinski V, Iadecola C, Petersen RC, Breteler MM, Nyenhuis DL, Black SE, Powers WJ, DeCarli C, Merino JG, Kalaria RN (2006). National Institute of Neurological Disorders and Stroke-Canadian Stroke Network vascular cognitive impairment harmonization standards. Stroke.

[16] Pendlebury ST, Cuthbertson FC, Welch SJ, Mehta Z, Rothwell PM (2010). Underestimation of cognitive impairment by Mini-Mental State Examination versus the Montreal Cognitive Assessment in patients with transient ischemic attack and stroke: a population-based study. Stroke.

[17] Popovic IM, Seric V, Demarin V (2007). Mild cognitive impairment in symptomatic and asymptomatic cerebrovascular disease. J Neurol Sci.

[18] Toglia J, Fitzgerald KA, O’Dell MW, Mastrogiovanni AR, Lin CD (2011). The Mini-Mental State Examination and Montreal Cognitive Assessment in persons with mild subacute stroke: relationship to functional outcome. Arch Phys Med Rehabil.

[19] Freitas S, Simoes MR, Alves L, Santana I (2011). Montreal Cognitive Assessment (MoCA): normative study for the Portuguese population. J Clin Exp Neuropsychol.

[20] Rossetti HC, Lacritz LH, Cullum CM, Weiner MF (2011). Normative data for the Montreal Cognitive Assessment (MoCA) in a population-based sample. Neurology.

[21] UK BioBank. Available at: www.ukbiobank.ac.uk. Accessed on Sept 25, 2012.

[22] German National Cohort (GNC) Consortium (2014). The German National Cohort: aims, study design and organization. Eur J Epidemiol.

[23] The Dallas Heart Study. Available at: http://www.utsouthwestern.edu/research/translational-medicine/doing-research/dallas-heart/index.html. Accessed on 25 Sept 2012.

[24] Multi-Ethnic Study of Atherosclerosis. Available at https://www.mesa-nhlbi.org/default.aspx. Accessed on 25 Sept 2012.

[25] Qiu C, Cotch MF, Sigurdsson S, Klein R, Jonasson F, Klein BEK, Garcia M, Jonsson PV, Harris TB, Eiriksdottir G (2009). Microvascular lesions in the brain and retina: The age, gene/environment susceptibility–Reykjavik study. Ann Neurol.

[26] Sabayan B, van Buchem MA, Sigurdsson S, Zhang Q, Harris TB, Gudnason V, Arai AE, Launer LJ. Cardiac Hemodynamics are Linked With Structural and Functional Features of Brain Aging: The Age, Gene/Environment Susceptibility (AGES)‐Reykjavik Study. J Am Heart Assoc 2015;4(1). 10.1161/JAHA.114.001294 PMC4330056 25628405

[27] DeCarli C, Massaro J, Harvey D, Hald J, Tullberg M, Au R, Beiser A, D’Agostino R, Wolf PA (2005). Measures of brain morphology and infarction in the framingham heart study: establishing what is normal. Neurobiol Aging.

[28] Weinstein G, Beiser AS, Decarli C, Au R, Wolf PA, Seshadri S (2013). Brain imaging and cognitive predictors of stroke and Alzheimer disease in the Framingham Heart Study. Stroke.

[29] Hofman A, Murad SD, Duijn CM, Franco OH, Goedegebure A, Arfan Ikram M, Klaver CCW, Nijsten TEC, Peeters RP, Stricker BHC (2013). The Rotterdam Study: 2014 objectives and design update. Eur J Epidemiol.

[30] Vermeer SE, Prins ND, den Heijer T, Hofman A, Koudstaal PJ, Breteler MM (2003). Silent brain infarcts and the risk of dementia and cognitive decline. N Engl J Med.

[31] Yan RT, Bluemke D, Gomes A, Burke G, Shea S, Liu K, Bahrami H, Sinha S, Wu C, Fernandes V (2011). Regional left ventricular myocardial dysfunction as a predictor of incident cardiovascular events MESA (multi-ethnic study of atherosclerosis). J Am Coll Cardiol.

[32] Friedrich MG, Larose E, Patton D, Dick A, Merchant N, Paterson I (2013). Canadian Society for CMR: Canadian Society for Cardiovascular Magnetic Resonance (CanSCMR) recommendations for cardiovascular magnetic resonance image analysis and reporting. Can J Cardiol.

[33] Hundley WG, Bluemke D, Bogaert JG, Friedrich MG, Higgins CB, Lawson MA, McConnell MV, Raman SV, van Rossum AC, Flamm S (2009). Society for Cardiovascular Magnetic Resonance guidelines for reporting cardiovascular magnetic resonance examinations. J Cardiovasc Magn Reson.

[34] Kwong RY, Chan AK, Brown KA, Chan CW, Reynolds HG, Tsang S, Davis RB (2006). Impact of unrecognized myocardial scar detected by cardiac magnetic resonance imaging on event-free survival in patients presenting with signs or symptoms of coronary artery disease. Circulation.

[35] Schelbert EB, Cao JJ, Sigurdsson S, Aspelund T, Kellman P, Aletras AH, Dyke CK, Thorgeirsson G, Eiriksdottir G, Launer LJ (2012). Prevalence and prognosis of unrecognized myocardial infarction determined by cardiac magnetic resonance in older adults. JAMA.

[36] Vanoverschelde JL, Wijns W, Depre C, Essamri B, Heyndrickx GR, Borgers M, Bol A, Melin JA (1993). Mechanisms of chronic regional postischemic dysfunction in humans. New insights from the study of noninfarcted collateral-dependent myocardium. Circulation.

[37] Vohringer M, Flewitt JA, Green JD, Dharmakumar R, Wang J, Tyberg JV, Friedrich MG (2010). Oxygenation-sensitive CMR for assessing vasodilator-induced changes of myocardial oxygenation. J Cardiovasc Magn Reson.

[38] Walcher T, Manzke R, Hombach V, Rottbauer W, Wohrle J, Bernhardt P (2012). Myocardial perfusion reserve assessed by T2-prepared steady-state free precession blood oxygen level-dependent magnetic resonance imaging in comparison to fractional flow reserve. Circ Cardiovasc Imaging.

[39] Bateman RJ, Xiong C, Benzinger TL, Fagan AM, Goate A, Fox NC, Marcus DS, Cairns NJ, Xie X, Blazey TM (2012). Clinical and biomarker changes in dominantly inherited Alzheimer’s disease. N Engl J Med.

[40] Hellings WE, Peeters W, Moll FL, Piers SR, van Setten J, Van der Spek PJ, de Vries JP, Seldenrijk KA, De Bruin PC, Vink A (2010). Composition of carotid atherosclerotic plaque is associated with cardiovascular outcome: a prognostic study. Circulation.

[41] Singh N, Moody AR, Rochon-Terry G, Kiss A, Zavodni A (2013). Identifying a high risk cardiovascular phenotype by carotid MRI-depicted intraplaque hemorrhage. Int J Cardiovasc Imaging.

[42] Britton KA, Massaro JM, Murabito JM, Kreger BE, Hoffmann U, Fox CS (2013). Body fat distribution, incident cardiovascular disease, cancer, and all-cause mortality. J Am Coll Cardiol.

[43] Foster MC, Hwang SJ, Porter SA, Massaro JM, Hoffmann U, Fox CS (2011). Fatty kidney, hypertension, and chronic kidney disease: the Framingham Heart Study. Hypertension.

[44] Fox CS, Gona P, Hoffmann U, Porter SA, Salton CJ, Massaro JM, Levy D, Larson MG, D’Agostino RB, O’Donnell CJ (2009). Pericardial fat, intrathoracic fat, and measures of left ventricular structure and function: the Framingham Heart Study. Circulation.

[45] Mahabadi AA, Massaro JM, Rosito GA, Levy D, Murabito JM, Wolf PA, O’Donnell CJ, Fox CS, Hoffmann U (2009). Association of pericardial fat, intrathoracic fat, and visceral abdominal fat with cardiovascular disease burden: the Framingham Heart Study. Eur Heart J.

[46] Preis SR, Massaro JM, Robins SJ, Hoffmann U, Vasan RS, Irlbeck T, Meigs JB, Sutherland P, D’Agostino RB, O’Donnell CJ (2010). Abdominal subcutaneous and visceral adipose tissue and insulin resistance in the Framingham heart study. Obesity.

[47] Rosito GA, Massaro JM, Hoffmann U, Ruberg FL, Mahabadi AA, Vasan RS, O’Donnell CJ, Fox CS (2008). Pericardial fat, visceral abdominal fat, cardiovascular disease risk factors, and vascular calcification in a community-based sample: the Framingham Heart Study. Circulation.

[48] Thanassoulis G, Massaro JM, O’Donnell CJ, Hoffmann U, Levy D, Ellinor PT, Wang TJ, Schnabel RB, Vasan RS, Fox CS (2010). Pericardial fat is associated with prevalent atrial fibrillation: the Framingham Heart Study. Circ Arrhythm Electrophysiol.

[49] Liu J, Fox CS, Hickson D, Bidulescu A, Carr JJ, Taylor HA (2011). Fatty liver, abdominal visceral fat, and cardiometabolic risk factors: the Jackson Heart Study. Arterioscler Thromb Vasc Biol.

[50] Liu J, Fox CS, Hickson DA, May WD, Hairston KG, Carr JJ, Taylor HA (2010). Impact of abdominal visceral and subcutaneous adipose tissue on cardiometabolic risk factors: the Jackson Heart Study. J Clin Endocrinol Metab.

[51] Liu J, Fox CS, Hickson DA, May WL, Ding J, Carr JJ, Taylor HA (2011). Pericardial fat and echocardiographic measures of cardiac abnormalities: the Jackson Heart Study. Diabetes Care.

[52] McAuley PA, Hsu FC, Loman KK, Carr JJ, Budoff MJ, Szklo M, Sharrett AR, Ding J (2011). Liver attenuation, pericardial adipose tissue, obesity, and insulin resistance: the Multi-Ethnic Study of Atherosclerosis (MESA). Obesity.

[53] Miao C, Chen S, Ding J, Liu K, Li D, Macedo R, Lai S, Vogel-Claussen J, Brown ER, Lima JA (2011). The association of pericardial fat with coronary artery plaque index at MR imaging: The Multi-Ethnic Study of Atherosclerosis (MESA). Radiology.

[54] Smith JD, Borel AL, Nazare JA, Haffner SM, Balkau B, Ross R, Massien C, Almeras N, Despres JP (2012). Visceral adipose tissue indicates the severity of cardiometabolic risk in patients with and without type 2 diabetes: results from the INSPIRE ME IAA study. J Clin Endocrinol Metab.

[55] Sidney S, Lewis CE, Hill JO, Quesenberry CP, Stamm ER, Scherzinger A, Tolan K, Ettinger B (1999). Association of total and central adiposity measures with fasting insulin in a biracial population of young adults with normal glucose tolerance: the CARDIA study. Obes Res.

[56] VanWagner LB, Wilcox JE, Colangelo LA, Lloyd-Jones DM, Carr JJ, Lima JA, Lewis CE, Rinella ME, Shah SJ (2015). Association of nonalcoholic fatty liver disease with subclinical myocardial remodeling and dysfunction: A population-based study. Hepatology.

[57] Chow CK, Lock K, Madhavan M, Corsi DJ, Gilmore AB, Subramanian SV, Li W, Swaminathan S, Lopez-Jaramillo P, Avezum A (2010). Environmental Profile of a Community’s Health (EPOCH): an instrument to measure environmental determinants of cardiovascular health in five countries. PLoS One.

[58] Corsi DJ, Subramanian SV, McKee M, Li W, Swaminathan S, Lopez-Jaramillo P, Avezum A, Lear SA, Dagenais G, Rangarajan S (2012). Environmental Profile of a Community’s Health (EPOCH): an ecometric assessment of measures of the community environment based on individual perception. PLoS One.

[59] Chiu M, Shah BR, Maclagan LC, Rezai MR, Austin PC, Tu JV (2015). Walk Score(R) and the prevalence of utilitarian walking and obesity among Ontario adults: A cross-sectional study. Health Rep.

[60] Statistics Canada. Longitudinal Survey of Immigrants to Canada*.* Available at: www23.statcan.gc.ca/imdb/p2SV.pl?Function = getSurvey&SDDS = 4422&lang = en&db = IMDB&dbg = f&adm = 8&dis = 2. Accessed Sept 25, 2012.

[61] Ryder AG, Alden LE, Paulhus DL (2000). Is acculturation unidimensional or bidimensional? A head-to-head comparison in the prediction of personality, self-identity, and adjustment. J Pers Soc Psychol.

[62] Tu JV, Donovan LR, Lee DS, Wang JT, Austin PC, Alter DA, Ko DT (2009). Effectiveness of public report cards for improving the quality of cardiac care: the EFFECT study: a randomized trial. JAMA.

[63] Tu JV, Chu A, Donovan LR, Ko DT, Booth GL, Tu K, Maclagan LC, Guo H, Austin PC, Hogg W (2015). The Cardiovascular Health in Ambulatory Care Research Team (CANHEART): using big data to measure and improve cardiovascular health and healthcare services. Circ Cardiovasc Qual Outcomes.

[64] Haroon NN, Austin PC, Shah BR, Wu J, Gill SS, Booth GL (2015). Risk of dementia in seniors with newly diagnosed diabetes: a population-based study. Diabetes Care.

[65] Targher G, Bertolini L, Rodella S, Tessari R, Zenari L, Lippi G, Arcaro G (2007). Nonalcoholic fatty liver disease is independently associated with an increased incidence of cardiovascular events in type 2 diabetic patients. Diabetes Care.

[66] Lazo M, Hernaez R, Bonekamp S, Kamel IR, Brancati FL, Guallar E, Clark JM (2011). Non-alcoholic fatty liver disease and mortality among US adults: prospective cohort study. BMJ.

[67] Taouli B, Serfaty L (2016). Magnetic Resonance Imaging/Elastography Is Superior to Transient Elastography for Detection of Liver Fibrosis and Fat in Nonalcoholic Fatty Liver Disease. Gastroenterology.

[68] Pencina MJ, D’Agostino RB, Demler OV (2012). Novel metrics for evaluating improvement in discrimination: net reclassification and integrated discrimination improvement for normal variables and nested models. Stat Med.

[69] McGorrian C, Yusuf S, Islam S, Jung H, Rangarajan S, Avezum A, Prabhakaran D, Almahmeed W, Rumboldt Z, Budaj A (2011). Estimating modifiable coronary heart disease risk in multiple regions of the world: the INTERHEART Modifiable Risk Score. Eur Heart J.

[70] Petersen SE, Matthews PM, Bamberg F, Bluemke DA, Francis JM, Friedrich MG, Leeson P, Nagel E, Plein S, Rademakers FE (2013). Imaging in population science: cardiovascular magnetic resonance in 100,000 participants of UK Biobank - rationale, challenges and approaches. J Cardiovasc Magn Reson.

[71] Hallal PC, Victora CG (2004). Reliability and validity of the International Physical Activity Questionnaire (IPAQ). Med Sci Sports Exerc.

[72] Kelemen LE, Anand SS, Vuksan V, Yi Q, Teo KK, Devanesen S, Yusuf S, Investigators S (2003). Development and evaluation of cultural food frequency questionnaires for South Asians, Chinese, and Europeans in North America. J Am Diet Assoc.

[73] Wechsler D (1958). The measurement and appraisal of adult intelligence.

[74] Borugian MJ, Robson P, Fortier I, Parker L, McLaughlin J, Knoppers BM, Bedard K, Gallagher RP, Sinclair S, Ferretti V (2010). The Canadian Partnership for Tomorrow Project: building a pan-Canadian research platform for disease prevention. CMAJ.

