# Supplementary material for: Rationale, design, and methods for Canadian alliance for healthy hearts and minds cohort study (CAHHM) – a Pan Canadian cohort study
Source: BMC Public Health. 2016 Jul 27;16:650. doi: 10.1186/s12889-016-3310-8 (PMC4963999; doi:10.1186/s12889-016-3310-8)
Supplement: Additional file 1: — CAHHM Local Site Investigators and MRI Coordination. CAHHM Participating Cohorts PIs. (DOCX 12 kb) [file 12889_2016_3310_MOESM1_ESM.docx]

**Additional file 1**

**CAHHM Local Site Investigators and MRI Coordination:**

**British Columbia:** Providence Health Care, Vancouver: Jonathan Leipsic (MRI); Simon Fraser University, Vancouver: Scott Lear

**Ontario:** Sunnybrook Health Sciences Centre, Toronto: Alan Moody

St. Michael’s Hospital, Toronto: Anish Kirpalani, David Jenkins, Andrew Yan, General Leung

Ottawa Heart Institute, Ottawa: Alexander Dick

Robarts Research Institute, London: Grace Parraga

St. Joseph’s Health Centre, Hamilton: Sonia Anand, Russell de Souza, Mike Noseworthy, Koon Teo

William Osler Health System, Brampton: David Kelton

**Alberta:** University of Calgary, Calgary: Eric Smith

University of Alberta, Edmonton: Ian Paterson

**Atlantic Provinces:**

Dalhousie University and IWK Health Centre, Halifax: Robert Miller, M Naeem Khan, Louise Parker, Matthias Schmidt, Trevor Dummer, David Thompson

**Quebec**:

Montreal Heart Institute/Geriatric Institute, Montreal: Francois Marcotte, Francois-Pierre Mongeon, Julie Robillard, Donato Terron, Anita Asgar

McGill University Health Centre, Montreal: Matthias Friedrich, Julie Lebel

Institut universitaire de cardiologie et de pneumologie de Québec, Université Laval, Quebec: Eric Larose, Jean-Pierre Despres, Paul Poirier, Gilles Dagenais,

**CAHHM Participating Cohorts PIs:**

**BC Generations Project:** John Spinelli (PI)

**Alberta’s Tomorrow Project:** Paula Robson (PI)

**Ontario Health Study:** Philip Awadalla (PI)

**CARTaGENE:** Sebastien Jacquemont (co-PI), Anne-Monique Nuyt (co-PI)

**Atlantic Path:** Louise Parker (PI), David Hoskin (Co-PI), Trevor Dummer (Academic Director)

**PURE- Canada:** Koon Teo (PI), Scott Lear (PI), Gilles Dagenais (PI), Salim Yusuf (PI)

**MHI BioBank:** Jean-Claude Tardif (PI), David Busseuil (Director)
